# Comment on Muth et al. Assessing Critical Flicker Fusion Frequency: Which Confounders? A Narrative Review. *Medicina* 2023, *59*, 800

**DOI:** 10.3390/medicina59091668

**Published:** 2023-09-15

**Authors:** Natalia D. Mankowska, Rita I. Sharma, Malgorzata Grzywinska, Anna B. Marcinkowska, Jacek Kot, Pawel J. Winklewski

**Affiliations:** 1 Applied Cognitive Neuroscience Laboratory, Department of Neurophysiology, Neuropsychology and Neuroinformatics, Medical University of Gdansk, 80-210 Gdansk, Poland; anna.marcinkowska@gumed.edu.pl; 2 Department of Neurophysiology, Neuropsychology and Neuroinformatics, Medical University of Gdansk, 80-210 Gdansk, Poland; rita.sharma@gumed.edu.pl (R.I.S.); pawel.winklewski@gumed.edu.pl (P.J.W.); 3 National Centre for Hyperbaric Medicine, Institute of Maritime and Tropical Medicine in Gdynia, Medical University of Gdansk, 80-210 Gdansk, Poland; jkot@gumed.edu.pl; 4 Neuroinformatics and Artificial Intelligence Laboratory, Department of Neurophysiology, Neuropsychology and Neuroinformatics, Medical University of Gdansk, 80-210 Gdansk, Poland; malgorzata.grzywinska@gumed.edu.pl; 5 2nd Department of Radiology, Medical University of Gdansk, 80-210 Gdansk, Poland

We first want to thank the authors of the excellent review for their contributions to summarizing the confounders associated with critical flicker fusion frequency (CFFF) [1]. We read this review with great interest as the publication significantly broadened with this aspect of the summary we wrote [2].

The main problem with flickering light is that there is still no clarity on how it is processed by the brain, and as a result, it is not clear what the results of a flicker test performed under different conditions actually show us [3]. As we pointed out in our other review [3], the processing of flickering light should be considered as a neurophysiological process as well as a neuropsychological one, and at this point, there is still no comprehensive explanation for these processes. There are papers identifying part of the issue (e.g., the colour of the flickering stimulus and its impact on brain activity or inducing epileptic seizures) [4,5], but there is a lack of explanation which would be relevant from the perspective of fully understanding CFFF.

Understanding these processes is even more important because flickering light is not only related to diving and medical diagnostics, but it is also closely related to the environment around us. It commonly appears mainly in electronic devices or lighting, which are as common as they are evolutionarily new, due to technologies that have been used by humans for a relatively short time.

Attempts are also being made to integrate CFFF into neuropsychological diagnosis. As indicated by Mewborn et al. [6] in their study, cited both by us and Muth et al. [1], the CFFF thresholds were correlated with scores on tests examining executive function. They pointed out that the sensory functions and processing speed, used in the flicker test, may be relevant to executive functions, but it is possible that the CFFF scores were correlated with stronger brain connectivity (as efficient executive functions also require this) [7]. As Muth et al. [1] justify, the reduction in retinal illumination may impact the lower CFFF threshold in older people compared to the young ones [8], but in view of explanations provided by Mewborn and colleagues [6], we might also expect that these differences were the result of brain connectivity deteriorating with age [9,10,11]. So what connectivity networks are involved in flickering light processing? We need to answer this question if we want to consider CFFF as a predictor of cognitive functioning.

As we described earlier [3], the processing of flickering light can be considered in accordance with signal detection theory, as also pointed out by Muth et al. [1], who indicated that it may explain differences in CFFF thresholds. In addition to the participant characteristics they highlighted, we must also consider the level of complexity of the stimuli used in the test, as it can affect the decision-making process for light characteristics [3].

It is necessary to establish the relationship between CFFF (and flickering light in general) and the aforementioned factors so that future research protocols will more accurately reflect the relationship between CFFF and the examined variables (e.g., arousal), rather than being determined by confounding factors that could be then better understood, eliminated, or controlled, including characteristics of the study participants or light.

We agree with Muth et al. [1] that the state of knowledge about CFFF still has many gaps and ambiguities. Possibly, an international cooperation involving key research teams exploring the intricacies of flickering light could represent an important step toward developing general protocol standards.

We believe that an understanding of the neurophysiological and neuropsychological processes underlying flickering light processing by the brain would help to clarify the role of the described confounders and thus to interpret them, particularly in the context of a very demanding underwater environment [12].
